# Неонатальный тиреотропный гормон — индикатор мониторинга тяжести йодного дефицита. Что считать «точкой отсечения»?

**DOI:** 10.14341/probl12892

**Published:** 2022-12-20

**Authors:** Л. А. Суплотова, О. Б. Макарова, Е. А. Трошина

**Affiliations:** Тюменский государственный медицинский университет; Тюменский государственный медицинский университет; Национальный медицинский исследовательский центр эндокринологии

**Keywords:** йодный дефицит, неонатальный ТТГ, мониторинг, скрининг, беременность, Тюменская область

## Abstract

ОБОСНОВАНИЕ. Вопросы мониторинга эффективности программ профилактики йодного дефицита (ЙД) являются важной составляющей в процессе ликвидации ЙД. Неонатальный тиреотропный гормон (ТТГ) используется в качестве критерия тяжести ЙД с 1994 г., однако вопрос о «точке отсечения» уровня неонатального ТТГ широко обсуждается в литературе последних лет.ЦЕЛЬ. Оценить критерий неонатальной гипертиреотропинемии выше 5 мМЕ/л с позиции мониторинга ЙД и установить «точку отсечения» на модели здоровых беременных женщин с адекватным йодным статусом.МАТЕРИАЛЫ И МЕТОДЫ. Проведено проспективное исследование в популяции беременных женщин г. Тюмени с формированием групп наблюдения по уровню экскреции йода с мочой — основная группа (с адекватным йодным статусом на протяжении всего периода гестации) и группа сравнения (женщины с показателями концентрации йода в моче (КЙМ) менее 150 мкг/л). Оценены результаты неонатального скрининга на врожденный гипотиреоз (ВГ) у детей женщин, участвовавших в исследовании. Проведена оценка частоты неонатального ТТГ выше 5 мМЕ/л в группах наблюдения. Выполнен ROC-анализ и установлена «точка отсечения» уровня неонатального ТТГ в качестве индикатора йодного дефицита.РЕЗУЛЬТАТЫ. Медианная КЙМ в популяции беременных женщин г. Тюмени составила 159,05 мкг/л, частота зоба — 0,38%, частота неонатальной гипертиреотропинемии выше 5 мМЕ/л — 2,88%, что характеризует адекватное йодное потребление в популяции беременных женщин. Частота неонатального ТТГ выше 5 мМЕ/л у новорожденных от женщин из основной группы составила 1,47%, а в группе сравнения — 9,3% (р=0,076). ROC-анализ выявил пороговое значение неонатального ТТГ 2,77 мМЕ/л в точке «cut-off», которому соответствовало наивысшее значение индекса Юдена. КЙМ более 150 мкг/л прогнозировалась при значении неонатального ТТГ ниже данной величины.ЗАКЛЮЧЕНИЕ. Анализ баз данных неонатального скрининга на ВГ позволяет эффективно, быстро и с минимальными затратами ежегодно проводить оценку йодного статуса в популяции. Установленная «точка отсечения» неонатального ТТГ на модели здоровых беременных с адекватным йодным потреблением в нашей работе — 2,77 мМЕ/л, отсутствие статистически значимых различий в частоте неонатальной гипертиреотропинемии выше 5 мМЕ/л у женщин с разным йодным статусом во время беременности свидетельствуют о необходимости пересмотра имеющегося порога 5 мМЕ/л и могут являться стимулом для проведения широкомасштабных исследований в регионах с разным йодным обеспечением.

## Abstract (en)

BACKGROUND: The issues of monitoring the effectiveness of iodine deficiency prevention programs are an important component in the process of iodine elimination. Neonatal thyrotropin (TSH) has been used as a criterion for the severity of iodine deficiency since 1994, however, the question of the “cut-off point” of the neonatal TSH level has been widely discussed in the recent literature.AIM: Evaluate the criterion for neonatal hyperthyroidism above 5 mIU/l from the perspective of monitoring iodine deficiency and establish a «cut-off point» on the model of healthy pregnant women with adequate iodine status.MATERIALS AND METHODS: A prospective study was conducted in a population of pregnant women in the city of Tyumen, with the formation of observation groups according to the level of iodine excretion in the urine — the main group (with adequate iodine status throughout the entire gestation period) and the comparison group (women with iodine levels less than 150 μg/l). The results of neonatal screening for congenital hypothyroidism in children of women participating in the study were evaluated. The frequency of neonatal TSH above 5mIU/l was assessed in the observation groups. ROC-analysis was performed and a «cut-off point» of the level of neonatal TSH was established as an indicator of iodine deficiency.RESULTS: The median urinary iodine concentration in the population of pregnant women in Tyumen was 159.05 μg/l, the incidence of goiter was 0.38%, the incidence of neonatal hyperthyroidism above 5 mIU/l was 2.88%, which characterizes adequate iodine intake in the pregnant population women. The frequency of neonatal TSH above 5 mIU/l in newborns from women from the main group was 1.47%, and in the comparison group — 9.3% (p = 0.076). ROC analysis revealed a threshold value of neonatal TSH of 2.77 mIU/l at the cut-off point, which corresponded to the highest value of the Youden index. Urinary iodine concentrations greater than 150 μg/l were predicted for nTSH values below this value.CONCLUSION: Analysis of databases of neonatal screening for congenital hypothyroidism makes it possible to effectively, quickly and at minimal cost annually assess the iodine status in the population. The established «cut-off point» of neonatal TSH in the model of healthy pregnant women with adequate iodine intake in our work is 2.77 mIU/l, the absence of statistically significant differences in the incidence of neonatal hyperthyroidism above 5 mIU/l from women with different iodine status during pregnancy indicate the need to revise the existing threshold of 5 mIU/l and may be an incentive to conduct large-scale studies in regions with different iodine supply.

## ОБОСНОВАНИЕ

Всемирная Организация Здравоохранения (ВОЗ) в 1994 г. включила уровень неонатального тиреотропного гормона (ТТГ) выше 5 мМЕ/л в индикаторы степени тяжести йодного дефицита (ЙД)1. Однако при литературном поиске в базах данных PubMed, Cochrane и документах ВОЗ на глубину 40 лет не найдены источники, которые лежат в основе расчета «точки отсечения» показателя неонатальной гипертиреотропинемии как индикатора тяжести ЙД. Вероятно, точка отсечения уровня неонатального ТТГ выше 5 мМЕ/л в качестве индикатора степени тяжести ЙД принята на основании мнения экспертов, научное обоснование которого не представлено. Данный вопрос возникает в связи с появлением публикаций, посвященных оценке тяжести ЙД по данному критерию с противоречивыми результатами [1–7]. Частота неонатальной гипертиреотропинемии в качестве индикатора мониторинга реализации профилактических программ имеет ряд преимуществ перед другими маркерами, характеризующими обеспеченность йодом (медианная концентрация экскреции йода с мочой (мКЙМ), частота зоба, уровень тиреоглобулина), так как скрининг на врожденный гипотиреоз (ВГ), на основании которого и рассчитывается данный показатель, охватывает всех новорожденных, проводится ежегодно с 1994 г. во всех регионах России, а анализ результатов не требует дополнительных финансовых затрат [8–10]. Однако на сегодняшний день «золотым стандартом» мониторинга ЙД остаются национальные или субнациональные исследования, включающие определение экскреции йода с мочой в целевых группах (дети препубертатного возраста (ДПВ), беременные и женщины репродуктивного возраста), которые, согласно рекомендациям ВОЗ, должны проводиться каждые 5 лет, наряду с определением содержания йода в соли, используемой в домохозяйствах. Все указанные мероприятия являются достаточно затратными даже для развитых стран и требуют немалых организационных усилий2 [10][11]. Весьма перспективным представляется использование результатов скрининга на ВГ с определением частоты неонатальной гипертиреотропинемии как для оценки выраженности дефицита йода, так и для понимания эффективности профилактических мер, направленных на его устранение. Все вышеизложенное, а также противоречивые результаты мониторинга в ряде стран, отсутствие исследований, позволяющих рассчитать «точку отсечения» уровня неонатального ТТГ, явились основанием для проведения данной работы.

## ЦЕЛЬ ИССЛЕДОВАНИЯ

Оценить критерий неонатальной гипертиреотропинемии выше 5 мМЕ/л с позиции мониторинга ЙД и установить «точку отсечения» на модели здоровых беременных женщин с адекватным йодным статусом.

## МАТЕРИАЛЫ И МЕТОДЫ

## Место и время проведения исследования

Место проведения. Исследование реализовано на базе ФГБОУ ВО «Тюменский ГМУ» Минздрава России, ГБУЗ ТО «Перинатальный центр» г. Тюмени, женская консультация №2 ГБУЗ ТО «Роддом №2».

Время исследования. Проспективный этап исследования проведен с июля 2019 г. по август 2020 г. Дополнительно выполнен ретроспективный анализ неонатального ТТГ по Тюменской области за период 1994–2020 гг.

## Изучаемые популяции (одна или несколько)

Изучались 2 популяции: беременные женщины, новорожденные.

## Беременные женщины включались в исследование в соответствии с критериями включения и исключения.

Критерии включения.

Критерии исключения.

Новорожденные — проведен анализ результатов неонатального ТТГ, полученных в рамках скрининга на ВГ детей, рожденных у женщин, принимавших участие в проспективном этапе исследования. Также проанализирована база данных неонатального ТТГ с 1994 по 2020 г. по Тюменской области (n=370 874).

## Способ формирования выборки из изучаемой популяции (или нескольких выборок из нескольких изучаемых популяций)

Для расчета «точки отсечения» неонатального ТТГ в качестве критерия мониторинга ЙД, по результатам завершенной беременности, женщины без патологии щитовидной железы были стратифицированы на 2 группы: основную (с адекватным йодным статусом на протяжении всего периода гестации) и группу сравнения (с недостаточным йодным статусом во время беременности).

## Дизайн исследования

Проведено одноцентровое проспективное исследование. На 1-м этапе проводился сплошной скрининг беременных женщин, в ходе которого в соответствии с критериями включения и исключения сформирована группа, вошедшая в проспективный 2-й этап исследования (рис. 1).

**Figure:**
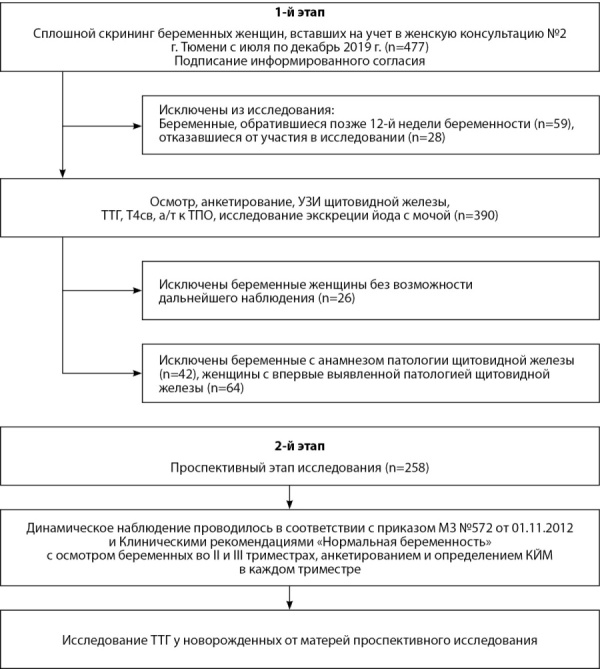
Рисунок 1. Дизайн исследования.Figure 1. Study design.

Из 477 беременных, вставших на учет в женской консультации с июля 2019 г. по декабрь 2019 г., 219 были исключены из анализа данных по причине несоответствия критериям включения и исключения: позднее обращение (n=59), патология щитовидной железы в анамнезе (n=42: субклинический гипотиреоз — 31, аутоиммунный тиреоидит (АИТ) — 4, узловой зоб — 3, гестационный тиреотоксикоз — 2, болезнь Грейвса в анамнезе — 1, гемиструмэктомия по поводу узлового зоба — 1), отказ от участия (n=28), невозможность дальнейшего наблюдения (n=26). По результатам обследования на этапе скрининга исключены 64 женщины с впервые выявленной патологией щитовидной железы и носительством антител к тиреопероксидазе (АТ-ТПО).

Все женщины были информированы о важности йодной профилактики во время беременности и грудного вскармливания, всем беременным назначались препараты йодида калия в физиологической дозе. Наблюдение беременных женщин велось в соответствии с приказом МЗ №572 от 01.11.20123 и клиническими рекомендациями «Нормальная беременность»4.

## Методы

Беременным, включенным в исследование, проводились анкетирование, позволяющее оценить использование йодированной соли в домохозяйствах, осмотр эндокринолога, включающий пальпаторное исследование щитовидной железы. Ультразвуковое исследование щитовидной железы проводилось с использованием УЗ-сканера 200 Pie Medical, датчиком с частотой 7,5 МГц с определением ее размеров и структуры.

Уровни тиреотропного гормона, свободной фракции тироксина (Т4св), АТ-ТПО определялись методом иммуноферментного анализа в клинико-диагностической лаборатории университетской многопрофильной клиники ФГБОУ ВО «Тюменский ГМУ» Минздрава России (зав. диагностическим отделением УМК ФГБОУ ВО «Тюменский ГМУ» Минздрава России к.м.н. Н.Ю. Южакова).

Определение уровня экскреции йода с мочой проводилось церий-арсенитовым методом в лаборатории клинической биохимии ФГБУ «НМИЦ эндокринологии» Министерства здравоохранения РФ (Москва) (директор — член-корр. РАН Н.Г. Мокрышева),с вычислением мКЙМ. Тяжесть ЙД оценивалась по критериям ВОЗ (2007)5. Исследование неонатального ТТГ проводилось в рамках скрининга на ВГ на базе ГБУЗ ТО «Перинатальный центр» (Тюмень) методом двустороннего флюорометрического иммуноферментного анализа с использованием наборов Delfia neonatal TSH (Percin Eimer, Финляндия).

## Статистический анализ

Статистический анализ проводился с использованием пакета программ Microsoft Office Excel 2010, STATISTICA 10 (StatSoft) и программы StatTech v. 1.2.0 (разработчик — ООО Статтех, Россия). Прогностическая модель, характеризующая зависимость количественной переменной от факторов, представленных количественными показателями, разрабатывалась с помощью метода парной или множественной линейной регрессии. Для оценки диагностической значимости неонатальной гипертиреотропинемии при прогнозировании наличия йодного дефицита или его отсутствия применялся метод анализа ROC-кривых. ROC-анализ позволяет с высокой точностью подобрать порог отсечения и выбрать модель с наилучшей прогностической силой. При ROC-анализе статистические модели считались приемлемыми, если они соответствовали следующим критериям: диагностическая чувствительность и специфичность более 50%, площадь под характеристической кривой (AUC, Area Under Curve) более 0,6 и порог статистической значимости, равный 0,05. Разделяющее значение количественного признака в точке «cut-off» определялось по наивысшему значению индекса Юдена. Различия оцениваемых показателей считались статистически значимыми при уровне p, равном 0,05.

## Этическая экспертиза

Проведение исследования одобрено Комитетом по этике при ФГБОУ ВО «Тюменский ГМУ» Минздрава России от 27 мая 2019 г. (выписка из протокола № 85).

## РЕЗУЛЬТАТЫ

По результатам обследования беременных на 1-м этапе частота зоба по УЗИ выявлена у 0,38% женщин, а медианная КЙМ составила 159,05 мкг/л, что соответствует адекватному йодному обеспечению в этой популяции (целевые показатели КЙМ 150–250 мкг/л). Частота неонатальной гипертиреотропинемии выше 5 мМЕ/л, известной для 312 новорожденных от матерей 1-го этапа скрининга, составила 2,88%. Таким образом, результаты исследования по всем критериям ВОЗ свидетельствуют об адекватном йодном потреблении в популяции беременных женщин г. Тюмени.

Для оценки доли беременных женщин проспективного этапа исследования с адекватным йодным статусом по триместрам выполнен анализ частотного распределения КЙМ (рис. 2).

**Figure:**
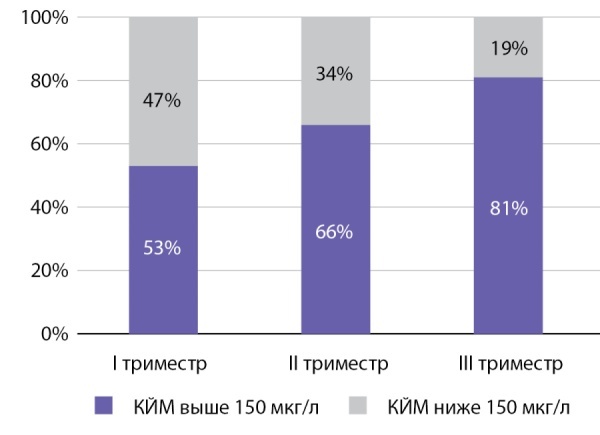
Рисунок 2. Доли беременных проспективного этапа исследования, имеющих показатели КЙМ выше и ниже целевого уровня 150 мкг/л, по триместрам беременности.Figure 2. Proportion of pregnant women in the prospective phase of the study with IM values above and below the target level of 150 µg/l by trimester of pregnancy.

По результатам анализа частотного распределения КЙМ установлено, что 47% беременных в I триместре имели показатели ниже 150 мкг/л. Как следствие информирования женщин о необходимости йодной профилактики в рамках консультации эндокринолога, акушера-гинеколога и прохождения школы беременных, доля женщин, имеющих адекватное йодное обеспечение, выросла с 53% в I триместре до 81% в III триместре. Несмотря на единый подход к ведению беременности, не все женщины выполняли рекомендации, о чем свидетельствуют показатели КЙМ и результаты анкетирования: йодированной солью пользовались на этапе включения в исследование только 55,2% женщин и 69,3% получали препараты, содержащие профилактическую дозу йода, к III триместру беременности доля беременных, использующих йодированную соль, возросла до 72,4, и до 84,32% увеличилось количество женщин, использующих препараты йода.

С целью оценки йодного статуса по критерию неонатального ТТГ выше 5 мМЕ/л проведен анализ результатов показателей ТТГ у новорожденных от матерей с разным йодным обеспечением в III триместре беременности, полученных в рамках неонатального скрининга на ВГ (табл. 1).

**Table:** Таблица 1. Частота неонатальной гипертиреотропинемии у детей в группах беременных женщин в г. Тюмени с разным йодным статусом в III триместре беременностиTable 1. The frequency of neonatal hyperthyrotropinemia in children in groups of pregnant women in the city of Tyumen, with different iodine status in the III trimester of pregnancy

	КЙМ выше 150 мкг/л (n=136)	КЙМ ниже 150 мкг/л (n=32)	р
Частота неонатального ТТГ выше 5 мМЕ/л	1, 47% (2)	9,3% (3)	0,076

Частота неонатального ТТГ выше 5 мМЕ/л у новорожденных от женщин с адекватным йодным обеспечением в III триместре составила 1,47% (у 2 детей из 136), что входит в целевые значения ниже 3%, рекомендованные ВОЗ, в то время как в группе беременных с КЙМ ниже 150 мкг/л в III триместре уровень неонатальной гипертиреотропинемии был равным 9,3% (у 3 из 32 детей).

Проведен анализ неонатального ТТГ (нТТГ) у новорожденных от женщин основной группы и группы сравнения, медиана и интерквартильный размах нТТГ представлены в таблице 2.

**Table:** Таблица 2. Показатели нТТГ у новорожденных от женщин основной группы и группы сравненияTable 2. Indicators of nTSH in newborns from women of the main and comparison groups Примечание. * — различия показателей статистически значимы (p<0,05).

Категории	Неонатальный ТТГ, мМЕ/л	p
Me	Q₁–Q₃
Группа сравнения КЙМ ниже 150 мкг/л	2,25	1,53–3,57	0,010*
Основная группа КЙМ выше 150 мкг/л	1,49	1,10–2,38

Медиана в основной группе составила 1,49 [ 1,10–2,38] мМЕ/л, в то время как в группе сравнения — 2,25 [ 1,53–3,57] мМЕ/л. Согласно полученным данным, при анализе нТТГ в основной группе и группе сравнения были установлены статистически значимые различия (p=0,010) (используемый метод: U–критерий Манна–Уитни).

Для расчета «точки отсечения» уровня нТТГ, свидетельствующего об адекватном йодном обеспечении женщин, применен метод анализа ROC-кривых (метод, позволяющий оценить диагностическую значимость нТТГ при прогнозировании определенного исхода, в нашем случае — КЙМ более 150 мкг/л, и использования любых методов профилактики ЙД).

При оценке зависимости вероятности КЙМ более 150 мкг/л в III триместре от уровня нТТГ с помощью ROC-анализа была получена следующая кривая (рис. 3).

**Figure:**
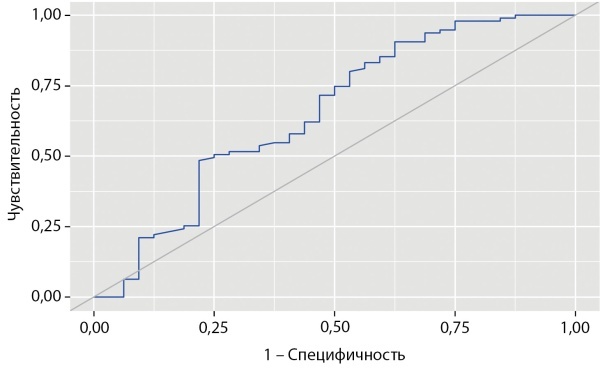
Рисунок 3. ROC-кривая, характеризующая зависимость вероятности показателя КЙМ выше 150 мкг/л в III триместре у беременных от уровня нТТГ.Figure 3. ROC-curve characterizing the dependence of the probability of an IMC indicator above 150 µg/l in the III trimester in pregnant women on the level of nTSH.

Площадь под ROC-кривой составила 0,652±0,059 с 95% доверительным интервалом 0,538–0,767. Полученная модель была статистически значимой (p=0,010).

Пороговое значение нТТГ в точке «cut-off», которому соответствовало наивысшее значение индекса Юдена, составило 2,77 мМЕ/л. КЙМ более 150 мкг/л прогнозировалась при значении нТТГ ниже данной величины. Чувствительность испецифичность модели составили 90,5 и 37,5% соответственно (рис. 4).

**Figure:**
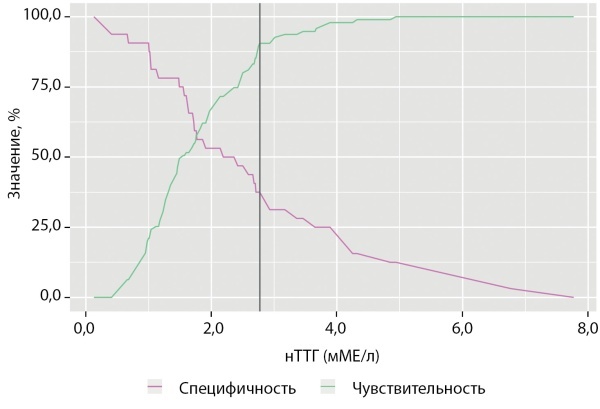
Рисунок 4. Анализ чувствительности и специфичности модели в зависимости от пороговых значений нТТГ.Figure 4. Sensitivity and specificity analysis of the model as a function of nTSH thresholds.

## ОБСУЖДЕНИЕ

В мире реализуются разные подходы и программы профилактики ЙД. Так, в большинстве стран внедрены программы всеобщего йодирования соли, в ряде стран используется йодирование продуктов питания в пищевой промышленности (по данным Всемирной сети по йоду, 236 стран имеют программы, и осталось 18 государств без внедрения всеобщего йодирования соли, в том числе и Россия)6 [12]. В России в целом на сегодняшний день реализуется добровольная модель профилактики ЙД, предоставляющая домохозяйствам возможность самостоятельно делать выбор в пользу йодированной соли, однако ряд территорий осуществляют региональные программы, которые включают использование йодированной соли при приготовлении пищи в образовательных учреждениях разного уровня, а в популяции беременных применяется групповая профилактика с использованием препаратов йода в физиологической концентрации [13–15]. Таким образом, в условиях реализации программ профилактики ЙД встает вопрос надежного индикатора мониторинга. Первоначально ВОЗ в 1994 г. были предложены несколько критериев оценки тяжести ЙД (табл. 3), и «золотым стандартом» до настоящего времени считается определение медианной концентрации КЙМ в группах ДПВ с целью как диагностики степени тяжести, так и мониторинга эффективности программ профилактики. Однако в связи с разными подходами к профилактике ЙД, в том числе в России, где с 2020 г. вступили в силу СанПиН 2.3/2.4.3590-20 «Санитарно-эпидемиологические требования к организации общественного питания населения»7, требующие обязательного использования йодированной соли для приготовления пищи в школьных столовых по всей стране, — определение КЙМ в группах ДПВ не будет отражать ситуацию в популяции как в целом, так и в группах беременных женщин, потребности в йоде которых выше. Кроме того, для выполнения таких исследований необходимы организация экспедиционных бригад, наличие сертифицированных лабораторий для выполнения исследования содержания йода в моче, что в целом является затратным для государства. Показатели частоты зоба в популяции школьников на сегодняшний день практически потеряли свою актуальность, так же как и уровень тиреоглобулина, который в качестве маркера ЙД не нашел широкого применения в мире [8][16]. В этих условиях использование показателей нТТГ, определяемого в рамках программы неонатального скрининга на ВГ, в качестве индикатора эффективности программ профилактики ЙД представляет большой интерес.

**Table:** Таблица 3. Индикаторы йододефицитных состояний по ВОЗ, 1994 и 2007 гг. [16]Table 3. WHO indicators of iodine deficiency, 1994 and 2007 [16] Примечание. * — дети пубертатного возраста

Индикатор	Референтная популяция	Степень тяжести йодного дефицита
легкая	умеренная	тяжелая
Медианная концентрация КЙМ, мкг/л	ДПВ*	50–99	20–49	<20
Зоб (увеличение щитовидной железы >0 степени), %	ДПВ	5–19,9	20–29,9	≥30
Тиромегалия (УЗ-объем >97 перцентиля >2 SDS), %	ДПВ	5–19,9	20–29,9	≥30
ТТГ цельной крови >5 мЕД/л, %	Новорожд.	3–19,9	20–39,9	≥40
Медиана тиреоглобулина, нг/мл, (сыворотка)	Дети/взрослые	10,0–19,9	20,0–39,9	≥40

Согласно рекомендациям ВОЗ, для территорий с благополучным йодным обеспечением уровень нТТГ выше 5 мМЕ/л определяется не более чем у 3% новорожденных, в регионах с легким ЙД — у 3–19,9%, с умеренным — у 20–39,9% детей [16]. В России скрининг на ВГ внедрен с 1994 г., проводится во всех регионах страны с охватом более 90% всех новорожденных, анализ результатов не требует дополнительных финансовых затрат, на примере Тюменской области динамика с 1994 по 2020 гг. демонстрирует значимое снижение доли новорожденных с уровнем нТТГ выше 5 мМЕ/л (рис. 5) [10][17].

**Figure:**
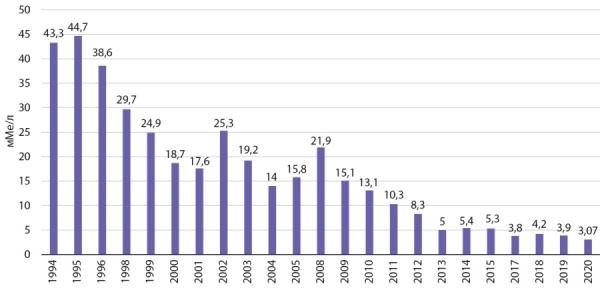
Рисунок 5. Частота неонатального ТТГ выше 5 мМЕ/л в Тюменской области (1994–2020 гг.).Figure 5. Frequency of neonatal TSH above 5 mIU/L in the Tyumen region (1994–2020).

Щитовидная железа новорожденного очень чувствительна к йодному статусу во время беременности, и предполагается, что даже легкий ЙД в этот период приведет к увеличению секреции нТТГ [13–15]. Таким образом, перспективным является рассмотрение вопроса оценки йодного статуса населения по частоте увеличения концентрации ТТГ у новорожденных выше 5 мМЕ/л в соответствии с рекомендациями ВОЗ [16]. И если по ЙД тяжелой и средней тяжести не возникает дискуссии в отношении показателей нТТГ выше 5 мМЕ/л, то в отношении легкого ЙД многочисленные работы последних лет демонстрируют противоречивые результаты. Так, широкомасштабные исследования в Бельгии показали, что частота нТТГ выше 5 мМЕ/л составляет 0,8–1,5% всех новорожденных, в то время как популяционные исследования КЙМ демонстрируют недостаточное йодное потребление в популяции беременных женщин [2]. Возможно, это отчасти объясняется использованием разных методик для определения нТТГ в стране (в Бельгии существует 6 центров скрининга, использующих 3 разных методики, но даже после применения поправочных коэффициентов доля новорожденных с ТТГ выше 5 мМЕ/л оставалась ниже 3%) [2]. В Австралии выявили только 2,2% новорожденных с уровнем ТТГ выше 5 мМЕ/л, несмотря на показатели мКЙМ 85 мкг/л [18]. В ряде других исследований, напротив, показано, что, несмотря на нормализацию йодного обеспечения, не происходит снижения частоты неонатальной гипертиреотропинемии ниже 3%. По результатам исследования в Грузии частота повышения нТТГ выше 5 мМЕ/л в целом по стране в 2013–2015 г. регистрировалась на уровне выше 3%, а в некоторых регионах — выше 5%. При этом вГрузии дефицит йода был преодолен еще в начале 2000-х годов, а мКЙМ составляла 242 и 226 мкг/л соответственно у школьников и беременных женщин [1]. Результаты последних исследований в Армении также продемонстрировали несоответствие показателей частоты неонатальной гипертиреотропинемии и йодного статуса населения — 3,44% при адекватном уровне КЙМ у школьников и у беременных женщин [19]. Однако при использовании фильтров, исключающих недоношенных, маловесных детей, а также уровни, превышающие 15 мМЕ/л, и те случаи, когда образцы крови были взяты вне рекомендованного интервала от 2 до 5 дней от даты рождения, были получены результаты, соответствующие критериям адекватного йодного статуса, — 2,86%.

Кроме того, в литературе последних лет широко обсуждается «точка отсечения» — «cut-off» нТТГ, разделяющая территории по степени йодного обеспечения для использования в качестве индикатора ЙД. Исследование уровней нТТГ в Северной Ирландии с 2003 по 2014 гг. показало, что пороговое значение выше 2 мМЕ/л может указывать на возникающий легкий дефицит йода, в то время как по критерию нТТГ выше 5 мМЕ/л территория является благополучной по йодному обеспечению [5].

В нашем исследовании отсутствие статистической значимости различий частоты неонатальной гипертиреотропинемии выше 5 мМЕ/л в группах женщин с разным йодным обеспечением (р=0,076) также может свидетельствовать в пользу пересмотра уровня нТТГ выше 5 мМЕ/л как точки отсечения для оценки легкого ЙД.

ROC-анализ на модели «здоровых беременных» с оптимальным йодным статусом выявил «точку отсечения» нТТГ 2,77 мМЕ/л. Доли новорожденных, имеющих уровень ТТГ выше 2 мМЕ/л (как предлагается рядом авторов) и выше 2,77 мМЕ/л, как рассчитано в нашем исследовании, представлены в таблице 4.

**Table:** Таблица 4. Частота неонатальной гипертиреотропинемии у детей от матерей с разными уровнями КЙМ в III триместреTable 4. The frequency of neonatal hyperthyroidism in children from mothers with different levels of IM in the third trimester

	КЙМ выше 150 мкг/л	КЙМ ниже 150 мкг/л	р
Частота неонатального ТТГ выше 5 мМЕ/л	1,47% (2)	9,3% (3)	р=0,076
Частота неонатального ТТГ выше 2 мМЕ/л	30,8% (42)	73,9% (17)	р=0,000
Частота неонатального ТТГ выше 2,77 мМЕ/л	15,44% (21)	40,62% (13)	p=0,003

Как видно из таблицы 5, при снижении порогового уровня ТТГ до 2 и 2,77 мМЕ/л появляется статистически значимая разница в частоте гипертиреотропинемии в группах беременных с разным йодным статусом, что также поднимает вопрос онеобходимости дополнительных исследований для определения порогового уровня ТТГ, характерного для легкого ЙД.

Частота неонатальной гипертиреотропинемии выше 5 и 2 мМЕ/л, по результатам скрининга в г. Тюмени за 2020 г. и по данным литературы, представлена в таблице 5.

**Table:** Таблица 5. Частота неонатальной гипертиреотропинемии выше 5 мМе/л и выше 2 мМЕ/л в разных странах мира [25]Table 5. The frequency of neonatal hyperthyroidism above 5 mIU/l and above 2 mIU/l in different countries of the world [25]

	Год	Частота неонатального ТТГ выше 5 мМЕ/л	Частота неонатального ТТГ выше 2 мМЕ/л
г. Тюмень, Россия	2020	3,07	31,18
Ирландия, Burns R. [3]	1995–2006	2,4–3,6	19,5–29,3
Бельгия, Vandevijvere S. [2]	2009–2011	2,6–3,3	21,0–40,6
Уэльс, Evans C [4]	2011–2013	0,9	10,9–11,9
Северная Ирландия, Mullan K. [5]	2003–2014	0,5	4,1–9,8

Результаты частоты неонатального ТТГ выше 2 мМЕ/л в популяции новорожденных Тюмени в 2020 г. сопоставимы с данными, полученными в Ирландии (19,5–29,3%) и Бельгии (21,0–40,6%) [2][3], однако в этих странах в популяции беременных женщин регистрировались низкие уровни КЙМ, свидетельствующие о йодном дефиците, а по результатам нашего исследования в популяции беременных женщин достигнуто оптимальное йодное потребление. Таким образом, вопрос о «точке отсечения» остается до настоящего времени открытым и требует дальнейшего изучения в регионах с разным йодным обеспечением.

## Ограничения исследования

Ограничением исследования является небольшая выборка популяции беременных.

## Направления дальнейших исследований

В продолжение, для отработки «точки отсечения» нТТГ как индикатора ЙД, будет целесообразно проведение анализа баз данных на ВГ в регионах с разным йодным статусом.

## ЗАКЛЮЧЕНИЕ

Результаты изучения динамики неонатальной гипертиреотропинемии выше 5 мМЕ/л в Тюменской области за 25-летний период подтверждают эффективность использования критерия с позиции мониторинга ЙД. Таким образом, анализ баз данных неонатального скрининга на ВГ позволяет эффективно, быстро и с минимальными затратами ежегодно проводить оценку йодного статуса в регионе.

Необходимость пересмотра порога уровня нТТГ выше 5 мМЕ/л в качестве индикатора оценки ЙД подтверждается отсутствием статистически значимых различий частоты неонатальной гипертиреотропинемии выше 5 мМЕ/л в группах женщин с разным йодным статусом и противоречивыми результатами зарубежных исследователей.

Установленная «точка отсечения» нТТГ на модели здоровых беременных с адекватным йодным потреблением в нашей работе — 2,77 мМЕ/л, а также результаты анализа порога неонатальной гипертиреотропинемии в 2 мМЕ/л во многих литературных источниках требуют обсуждения, также указывают на необходимость пересмотра уровня нТТГ 5 мМЕ/л в качестве критерия ЙД и могут являться стимулом для проведения широкомасштабных исследований в регионах с разным йодным обеспечением.

## ДОПОЛНИТЕЛЬНАЯ ИНФОРМАЦИЯ

Источники финансирования. Работа выполнена по инициативе авторов без привлечения финансирования

Конфликт интересов. Авторы декларируют отсутствие явных и потенциальных конфликтов интересов, связанных с содержанием настоящей статьи.

Участие авторов. Суплотова Л.А., Макарова О.Б. и Трошина Е.А. внесли значимый вклад в проведение исследования и подготовку статьи, а также одобрили финальную версию статьи перед публикацией, выразили согласие нести ответственность за все аспекты работы, подразумевающую надлежащее изучение и решение вопросов, связанных с точностью или добросовестностью любой части работы.

Благодарности. Авторы выражают благодарность региональному координатору Международной неправительственной организации «Глобальная сеть по йоду» по странам Восточной Европы и Центральной Азии (Мертл Бич, США), д.м.н., профессору Григорию Анатольевичу Герасимову за обсуждение результатов исследования, выделение акцентов и поддержку.

1. World Health Organization, International Council for Control of Iodine Deficiency Disorders & United Nations Children’s Fund (UNICEF). (1994). Indicators for assessing iodine deficiency disorders and their control through salt iodization. World Health Organization. https://apps.who.int/iris/handle/10665/70715 2. World Health Organization/International Council for the Control of the Iodine Deficiency Disorders/United Nations Childrens Fund (WHO/ICCIDD/UNICEF). Assessment of the iodine deficiency disorders and monitoring their elimination. Geneva: World Health Organization, 2007. Режим доступа: https://apps.who.int/iris/bitstream/handle/10665/43781/9789241595827_eng.pdf?sequence=1&isAllowed=y 3. Приказ Минздрава России от 01.11.2012 N 572н (ред. от 12.01.2016) «Об утверждении Порядка оказания медицинской помощи по профилю “акушерство и гинекология” (за исключением использования вспомогательных репродуктивных технологий» (Зарегистрировано в Минюсте России 02.04.2013 N 27960). Режим доступа: https://dr-fomin.ru/wp-content/uploads/2019/12/%D0%9F%D1%80%D0%B8%D0%BA%D0%B0%D0%B7-%D0%9C%D0%97-%D0%A0%D0%A4-N572%D0%BD.pdf 4. Долгушина Н.В., Артымук Н.В., Белокриницкая Т.Е. и др. Клинические рекомендации Российского общества акушеров-гинекологов. Нормальная беременность. 2019. Режим доступа: https://disk.yandex.ru/i/6WWXSxDEH7sjow 5. World Health Organization/International Council for the Control of the Iodine Deficiency Disorders/United Nations Childrens Fund (WHO/ICCIDD/UNICEF). Assessment of the iodine deficiency disorders and monitoring their elimination. Geneva: World Health Organization, 2007. Режим доступа:https://apps.who.int/iris/bitstream/handle/10665/43781/9789241595827_eng.pdf?sequence=1&isAllowed=y 6. Режим доступа: https://www.ign.org/cm_data/IGN_Global_Scorecard_MAP_2021_SAC_-_7_May_2021.pdf 7. Режим доступа: https://docs.cntd.ru/document/566276706
